# Doege-Potter syndrome presenting with hypoinsulinemic hypoglycemia in a patient with a malignant extrapleural solitary fibrous tumor: a case report

**DOI:** 10.1186/1752-1947-7-11

**Published:** 2013-01-09

**Authors:** Robert C Schutt, Trish A Gordon, Ruchi Bhabhra, Helen P Cathro, Stephen L Cook, Christopher R McCartney, Geoffrey R Weiss

**Affiliations:** 1 Department of Internal Medicine, University of Virginia, PO Box 800696, Charlottesville, VA, 22908, USA; 2 Department of Internal Medicine, Division of Endocrinology and Metabolism, University of Virginia, Charlottesville, USA; 3 Department of Pathology, University of Virginia, Charlottesville, USA; 4 Department of Internal Medicine, Division of Hematology and Oncology, University of Virginia, Charlottesville, USA

## Abstract

**Introduction:**

Doege-Potter syndrome is a paraneoplastic syndrome characterized by non-islet cell tumor hypoglycemia secondary to a solitary fibrous tumor. This tumor causes hypoglycemia by the secretion of a prohormone form of insulin-like growth factor II. We describe the diagnosis and management of Doege-Potter syndrome and the use of transarterial chemoembolization in a patient with a malignant extrapleural solitary fibrous tumor.

**Case presentation:**

Our patient was a 64-year-old Caucasian woman who initially presented with urinary incontinence and was found to have a 14.5×9.0×9.0cm retroperitoneal solitary fibrous tumor compressing her bladder. Her tumor was surgically resected but recurred with multiple hepatic metastatic lesions. The hepatic metastases progressed despite systemic chemotherapy and treatment with doxorubicin transarterial chemoembolization. Her course was complicated by the development of recurrent fasting hypoglycemia, most likely secondary to Doege-Potter syndrome. Her hypoglycemia was managed with corticosteroid therapy and frequent scheduled nutrient intake overnight.

**Conclusions:**

The rarity of hepatic solitary fibrous tumors and consequent lack of controlled trials make this report significant in that it describes the diagnostic approach to Doege-Potter syndrome, describes our experience with the use of doxorubicin transarterial chemoembolization, and presents management options for tumor-associated hypoglycemia in the case of extensive disease not amenable to surgical resection.

## Introduction

Doege-Potter syndrome [1,2] is a paraneoplastic syndrome characterized by non-islet cell tumor hypoglycemia (NICTH) secondary to a solitary fibrous tumor (SFT) that secretes a prohormone form of insulin-like growth factor II (IGF-II). Doege-Potter syndrome is an uncommon complication of an uncommon tumor. However, it is potentially life-threatening, and thus requires a careful and attentive approach to diagnosis and management. The following describes the diagnosis and management of Doege-Potter syndrome in a patient with an extrapleural solitary fibrous tumor (ESFT) complicated by hepatic metastases.

## Case presentation

**Figure 1:**
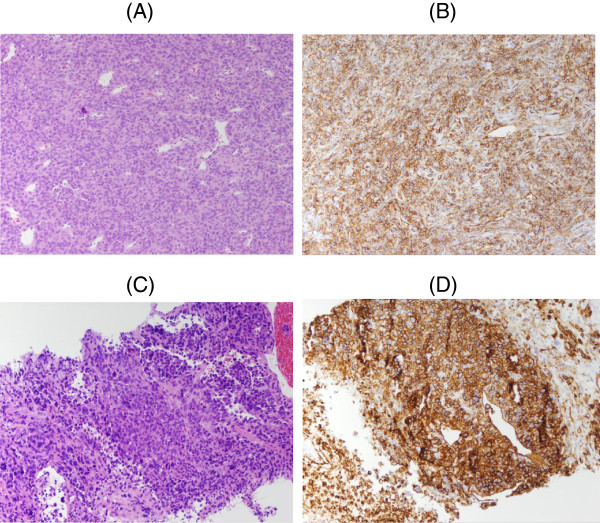
**Selected images from the pathological evaluation. (A)** The retroperitoneal mass is composed of cellular fusiform cells with a staghorn vascular pattern. Mitotic figures are frequent, but no atypical forms are seen (hematoxylin and eosin stain 10×). (**B**) An immunohistochemical stain for CD34 performed on the retroperitoneal mass (10×). (**C**) A core biopsy of the liver shows increased cellularity and increased cellular pleomorphism, with areas of necrosis (hematoxylin and eosin stain 10×). (**D**) An immunohistochemical stain for CD34 performed on liver mass (10×).

Our patient is a 64-year-old Caucasian woman who initially presented with urinary incontinence. She was found to have a 14.5×9.0×9.0cm retroperitoneal pelvic mass compressing her bladder. She did not have symptoms of hypoglycemia at this time. Our patient subsequently underwent exploratory laparotomy with resection of the retroperitoneal mass, sigmoidectomy and colostomy. On gross examination, the 14.5cm retroperitoneal mass was well-encapsulated with only central focal areas of necrosis. On microscopic examination, the tumor demonstrated increased cellularity, mildly pleomorphic epithelioid cells and 15 mitotic figures per 10 high power fields. Atypical mitotic figures were not appreciated. By contrast, the liver metastases were partially necrotic, and were composed of moderately pleomorphic sarcomatoid cells with far more numerous mitotic figures. Immunohistochemical staining of the spindle cells was strongly positive for cluster of differentiation (CD)-34 and B-cell lymphoma-2, with patchy positivity for vimentin. The tumor cells were negative for CD-99, estrogen receptor protein, CD-10, CD-117, muscle-specific actin, desmin, smooth muscle actin, epithelial membrane antigen, CD-31, keratin, S-100, inhibin and CD-56. The diagnosis of ESFT was made based on the microscopic and immunohistochemical findings. Selected images from the pathological evaluation are presented in Figure 1.

Our patient was followed-up with serial imaging, first at three months after resection, then at 15 months post-resection. On the latter scan, four new hepatic masses were identified: an 8.3×10.4cm mass within hepatic segment two, a 4.4×4.8cm mass within hepatic segment four, a 6.8×5.5cm lesion within hepatic segment eight, and a 2.2×2.2cm lesion within hepatic segment six. Fine needle aspiration of the largest lesion demonstrated a similar immunohistochemical profile to the primary retroperitoneal ESFT, and the newly identified masses were considered metastatic malignant ESFT. At this time, she was started on the multi-targeted receptor tyrosine kinase inhibitor sunitinib for treatment of the hepatic metastases. However, this was discontinued three months later due to the development of erythema multiforme, hyponatremia and thrombocytopenia. Given this sunitinib intolerance, she was treated with temozolomide and intravenous bevacizumab for four months. This regimen was tolerated well, but subsequent imaging demonstrated progression of the disease. The liver lesions were then treated with transarterial chemoembolization (TACE) using doxorubicin beads on two separate occasions - at 22 months and 24 months after initial diagnosis and resection. Despite chemoembolization, the liver lesions continued to progress in size, and a new lesion developed.

Approximately 22 months after her initial diagnosis (prior to the first TACE procedure) our patient developed symptomatic hypoglycemia. Her initial episode was heralded by a period of disorientation lasting approximately one hour. Her blood glucose was documented to be 41mg/dL at this time. These symptoms progressed to frequent overnight hypoglycemia requiring frequent snacking to maintain a normal blood glucose level. During the second chemoembolization procedure, she had persistent hypoglycemia (less than 40mg/dL), likely related to pre-procedural fasting. Reversal of hypoglycemia during the procedure required administration of six 25g ampules of 50% dextrose. Our patient was subsequently admitted to the hospital for evaluation of her hypoglycemia. During hospitalization, her blood glucose level decreased precipitously multiple times. Our patient’s hypoglycemia occurred primarily with fasting and was ameliorated with continuous dextrose 10% infusion and frequent snacks (for example, liquid nutritional supplements).

A laboratory evaluation was performed while our patient was hypoglycemic (blood glucose 26mg/dL), and pertinent results are listed in Table 1. Notably, she had low insulin and C-peptide concentrations in the setting of hypoglycemia, which effectively excluded insulinoma, ectopic insulin production or exogenous insulin administration as the cause of hypoglycemia. IGF-II and IGF-I was measured, and the IGF-II:IGF-I ratio was elevated at 9.60 (3:1 is normal, and >10 has been suggested to be pathognomonic for NICTH) [3]. In addition, a large sized tumor (>10cm) in the setting of hypoinsulinemic hypoglycemia indicated NICTH as the etiology for her hypoglycemia [4]. Thus, paraneoplastic Doege–Potter syndrome [5] was considered the most likely etiology of this patient’s hypoglycemia.

**Table 1:** Laboratory evaluation for hypoglycemia

**Test**	**Result**	**Reference range**
Glucose	26mg/dL	74 to 99mg/dL
Insulin	<2.0μIU/mL	<20μIU/mL
Proinsulin	2.7pmol/L	3 to 20pmol/L
C-peptide of insulin	<0.10ng/mL	0.50 to 2.00ng/mL
Beta-hydroxybutyrate	<0.1mmol/L	<0.4mmol/L
Insulin-like growth factor I	35ng/mL (4.59nmol/L)^a^	55 to 225ng/mL
Insulin-like growth factor II	331ng/mL (44.02nmol/L)^a^	288 to 736ng/mL
Adrenocorticotropic hormone	20pg/mL	9 to 52pg/mL
Cortisol	14.4μg/dL at 6:59 a.m.	at 8:00 a.m.: 4 to 19μg/dL
Glucagon stimulation test	Before glucagon: 26mg/dL	>30mg/dL
	10 minutes: 33mg/dL	
	20 minutes: 44mg/dL	
	30 minutes: 54mg/dL	
	Total Δ: 28mg/dL	

^a^Molar concentrations were calculated based on conversion factor of 0.131 for IGF-I and 0.133 for IGF-II.

**Figure 2:**
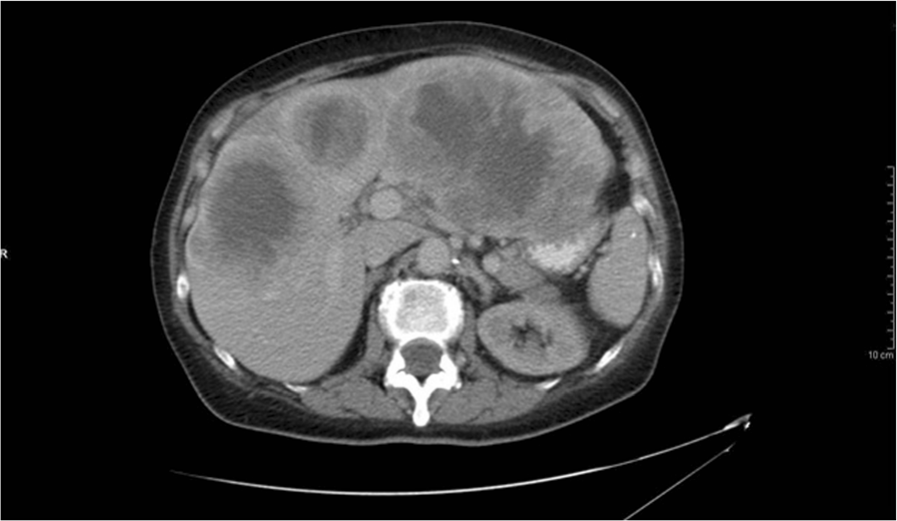
Computed tomography scan of the abdomen demonstrating metastatic solitary fibrous tumor involvement of the liver.

Extensive damage (replacement) of liver parenchyma with reduced hepatic glucose production was considered, as our patient had significant hepatic metastases. One milligram of glucagon was administered when her venous blood glucose level was 26mg/dL; 30 minutes after glucagon administration, her blood glucose had only risen to 54mg/dL (increment of 28mg/dL). Limited data suggest that an increment of less than or greater than 30mg/dL may distinguish patients with reduced hepatic glucose production from those with excessive insulin (or insulin-like) action, respectively [6]. Of interest, it is said that over 80% of the hepatic mass must typically be destroyed before this alone would cause fasting hypoglycemia [7], and although there was significant tumor involvement of the liver, imaging in our patient suggested that at least 20% normal liver remained (Figure 2). She also had evidence of normal synthetic function with an international normalized ratio of 1.2 (reference range 0.9 to 1.2). However, it is certainly possible that concurrent abnormalities (for example, malnutrition) in combination with her hepatic metasteses may have contributed to reduced hepatic glucose production and this may have manifested before detection of reduced hepatic synthetic capacity. With all available data taken together, we felt that reduced hepatic mass may have contributed to our patient’s fasting hypoglycemia, but it was unlikely to be the primary cause.

Hypoglycemia in this patient was most prominent overnight when her food intake decreased, and she had life-threatening nocturnal hypoglycemia on a number of occasions. Therefore, management of Doege-Potter syndrome in our patient was focused on maintaining euglycemia overnight. Potential management strategies are described in Table 2. Treatment options were discussed at length with our patient, and the decision was made to pursue non-invasive management strategies first. Initial management included scheduled, intermittent ingestion of liquid nutritional supplements overnight, triggered every three hours by an automatic alarm. Each liquid nutritional supplement (Ensure Plus®) contained 350 kilocalories, 50g of carbohydrate, 11g of fat and 13g of protein. In addition, 10mg of oral prednisone was administered daily [8]. This regimen appeared to prevent overnight hypoglycemia in the hospital, and our patient was discharged on 10mg oral prednisone to be taken each morning along with supplemental liquid nutrition to be taken consistently at 10:00 p.m., 1:00 a.m. and 4:00 a.m. On clinic follow-up two weeks later, her appetite had improved, she had gained three pounds, and she had well-controlled blood glucose levels all day with frequent snacks. Overnight hypoglycemia was less frequent, although her blood glucose levels periodically decreased into the 30s and 40s mg/dL, especially around 1:00 a.m. and between 5:00 and 7:00 a.m. She had not adhered to the scheduled liquid nutritional supplements, although she was eating throughout the night. Her prednisone regimen was changed to 7.5mg in the morning and 2.5mg in the evening. To help achieve steady blood glucose levels over longer periods of time, we also recommended eating snacks with raw cornstarch, which is relatively slowly digested by pancreatic amylase [9,10].

**Table 2:** Management strategies for Doege-Potter syndrome

**Strategy**	**Advantages**	**Disadvantages**
Surgically remove underlying tumor	Most solitary fibrous tumors are benign and adequate resection resolves hypoglycemia	Invasive; may not be a viable option (for example, malignant tumors with metastasis)
Systemic or localized chemotherapy	May be used to treat non-resectable tumors	Chemotherapy regimens are not well studied; significant side effects associated with chemotherapy; tumors are typically poorly responsive to systemic chemotherapy
Scheduled snacks	Non-invasive	Relies on patient adherence to schedule
Nocturnal or continuous dextrose infusion	Reliably prevents hypoglycemia	Requires long-term venous access with attendant risks (for example, infection)
Nocturnal or continuous enteral tube feeding	Reliably prevents hypoglycemia	Long-term use requires invasive placement of gastrostomy tube
Corticosteroid administration	Non-invasive; may normalize insulin-like growth factor levels; increases appetite	Multiple adverse effects of long-term corticosteroid use
Continuous glucagon infusion [6]	Effective to prevent hypoglycemia in some patients; subcutaneous administration has less infectious risk than direct venous access	May be practically difficult

Unfortunately, our patient had rapid clinical deterioration shortly thereafter. Her appetite decreased significantly, she developed marked ascites, and she was admitted to another hospital with failure to thrive, atrial fibrillation and hypotension. She was discharged home with hospice care and passed away two days later. Due to her rapid clinical course, she was not able to start the raw cornstarch diet, and she continued to have episodic overnight hypoglycemia until her death.

## Discussion

SFTs are rare spindle cell neoplasms first described clinically in 1870 by Wagner [11] and subsequently described by histopathology by Klemperer and Rabin in 1931 [12]. SFTs often derive from the pulmonary pleura, but they may also occur outside the pleura as ESFTs. ESFTs are rare soft tissues tumors; in one large retrospective series involving over 4000 soft tissue tumors obtained over 18 years, only 79 SFTs were identified, 54 of which were intrathoracic in origin [13]. Doege-Potter syndrome [1,2] is a paraneoplastic condition defined by NICTH secondary to a SFT; this occurs in less than 5% of SFT cases [14]. Table 3 presents a list of prior cases of Doege-Potter syndrome. The typical mechanism of hypoglycemia with SFTs involves tumor production of a prohormone form of IGF-II, often called ‘big IGF-II’ [15]. Big IGF-II causes hypoglycemia through multiple mechanisms [6,16,17]. Insulin-like effects of big IGF-II lead to increased glucose uptake by insulin-sensitive tissues, especially muscle and fat, but it may also stimulate glucose uptake by the tumor itself. Decreased hepatic glucose production may also be related to the insulin-like effect of big IGF-II, and glucagon secretion may be low in NICTH as well.

**Table 3:** Summary of prior cases of Doege-Potter syndrome

**Author**	**Year**	**Case information**
Doege [1]	1930	The patient presented with facial flushing and symptoms of irrationality described as ‘maniacal seizures’ noted to improve with treatment by rectal glucose, morphine and scopolamine. Symptoms continue to recur and urine analysis demonstrated traces of acetone. The patient was found to have 26×16cm tumor filling nearly the entire left thoracic cavity, and was treated with surgical excision and noted to be without recurrence at a three-year follow-up.
Potter [2]	1930	The patient was found to be delirious at times and was found to have a large tumor filling the entire left side of the thoracic cavity. The patient subsequently underwent two-stage surgical resection five days apart and made an uneventful recovery.
Arkless [18]	1942	The patient had a history of convulsive seizures and was found to have a left subphrenic mass. Removal resolved the seizures. Seven years later the patient presented with hypoglycemia. This was managed on a high carbohydrate diet and the patient died. A spindle cell sarcoma of the left chest was found postmortem.
Hines [19]	1943	A patient with recurrent hypoglycemia was found to have a sarcoma of the right upper abdomen.
Skillern *et al.*[20]	1954	The patient had two large tumors composed of round and spindled cells and was found to have recurrent hypoglycemia.
Frantz and Porter [21]	1956	The patient presented with recurrent hypoglycemia and was found to have a large pelvic mass encasing the ovary, thought to be a spindle cell neoplasm.
Holten [22]	1957	A patient with a spindle cell tumor causing hypoglycemia.
Scholz *et al*. [23]	1957	Two cases of patients who presented with recurrent hypoglycemia. In one patient, surgical resection of the tumor (described as a renal fibrosarcoma) resolved the hypoglycemia.
August and Hiatt [24]	1958	Patient with frequent episodes of hypoglycemia that ceased with surgical resection of a large intrathoracic fibrosarcoma.
Grilliat *et al.*[25]	1970	Tumor described as a pleural mesothelioma associated with hypoglycemia.
Ellorhaoui and Graf [26]	1976	Patient with an intrathoracic tumor found to have hypoglycemia and diagnosed with Doege-Potter syndrome.
Vollmar and Wockel [27]	1977	Tumor of the mesenchyme in the renal pelvis with malignant spread to lymph nodes and pathologically classified as malignant histiocytoma.
Payne and Davison [28]	1979	Intrathoracic spindle cell tumor.
Kecskés *et al*. [29]	1979	Thoracic mesenchymal tumor treated with surgical resection.
Dao *et al*. [30]	1984	Pleural tumor in a patient with hypoglycemia.
Heinrich *et al*. [31]	1984	Tumor described as an intrathoracic fibroma.
Lessel and Erbstosser [32]	1984	Malignant fibrous histocytoma of the right lung that infiltrated the spinal column causing paralysis, no metastatic disease identified.
Roy *et al.*[33]	1992	Recurrent SFT of the pleura.
Abonyi *et al.*[34]	1992	Left-sided pleural mesothelioma and electron microscopy demonstrated neurosecretory granules thought to be insulin-like growth factor II-like material.
Gullo *et al.*[8]	1999	Abdominal hemangiopericytoma treated with resection, developed recurrence that was treated with chemotherapy and radiotherapy, with subcutaneous biosynthetic growth hormone and prednisone to mitigate symptoms.
Chamberlain and Taggart [5]	2000	Tumor described as a sub-pleural fibroma measuring 23×21×12cm, treated with surgical resection and noted to have complete recovery.
Herrmann *et al*. [35]	2000	Malignant fibrous histiocytoma of the lung with tumor size described as 13×8cm.
Zafar *et al*. [36]	2003	Pleural SFT measuring 19×15×14cm.
Kafih *et al.*[37]	2005	Pleural fibrous tumor involving entire left hemithorax.
Balduyck *et al*. [38]	2006	Pleural SFT measuring 22×19×7cm.
Lucas and Ledgerwood [39]	2006	Malignant SFT of small bowel mesentery, measuring 10×12×20cm.
Hirai[40]	2006	Pleural SFT measuring 10.9×9.8×9.4cm treated with surgical resection, found to have corresponding resolution of hypoglycemia associated with decrease in serum insulin-like growth factor II level postoperatively.
Milenković *et al*. [41]	2007	Benign SFT involving almost the entire hemithorax.
Kalebi *et al*. [42]	2009	Pleural SFT measuring 20×15×10cm, treated with surgical resection.
Lee *et al*. [43]	2010	Thoracic SFT described pathologically as a low malignant potential tumor.
Fung and Crook [44]	2011	Patient with tumor associated with spontaneous hypoglycemia.
Campos *et al.*[45]	2012	Pleural SFT measuring 27×25×11.5cm.
Rosseel *et al*. [46]	2012	Patient with hypoglycemia and found to have a right thoracic mass.
Herrak *et al*. [47]	2012	Doege-Potter syndrome diagnosed in patient with a pleural tumor.

SFT: solitary fibrous tumor.

IGF-II assay results are frequently in the normal reference range in NICTH, and assays for big IGF-II are not commercially available. However, the IGF-II:IGF-I molar ratio is considered to be a surrogate marker of big IGF-II concentration [17]. In NICTH, IGF-II provides central negative feedback of growth hormone, with a subsequent reduction of IGF-I production. This contributes to an increase in the IGF-II:IGF-I ratio; a ratio of 3:1 is considered normal, with a ratio greater than 10 reported to be virtually pathognomonic for NICTH [3]. Parenthetically, decreased growth hormone secretion also leads to lower concentrations of IGF-binding proteins, which may increase the bioavailability of IGF-II [16].

Our case highlights the importance of considering Doege-Potter syndrome in a patient with SFT and hypoglycemia. This is especially important when the tumor is large (larger than 8cm to 10cm), as this is thought to be a risk factor for the development of this paraneoplastic syndrome [14,36]. In our case, the presence of massive liver metastases with extensive liver replacement complicated the precise determination of the etiology. The possibility of markedly reduced hepatic glucose output (for example, reduced glycogen stores) was considered; for reasons described previously, this was not felt to be the primary cause of our patient’s hypoglycemia, although it likely contributed. Liver replacement may also have been responsible for an IGF-II:IGF-I ratio less than 10, as shown in a case series of patients with NICTH secondary to a combination of liver destruction and excessive secretion of big-IGF-II, in which the patients had a lower than expected IGF-II:IGF-I ratio [4].

Treatment options for Doege-Potter syndrome can be divided into therapies that directly reduce tumor burden (for example, surgery, chemotherapy) and therapies that mitigate hypoglycemia without treating the underlying tumor. Our patient’s hepatic metastases exhibited no clear response to employed treatments. TACE has been used for treatment of ESFTs that are not surgically resectable, but there are few data regarding TACE for hepatic ESFT [47]. This in part reflects its rarity; a recent review summarizing treatment approaches to hepatic ESFT found only 30 cases of hepatic ESFT [48]. TACE did not appear to be effective in our patient, as follow-up imaging one month after the second TACE procedure demonstrated significant progression of disease. This is in contrast to a prior case report that demonstrated imaging evidence of response and a stable tumor size in a patient with a SFT treated with doxorubicin chemoembolization [49].

For our patient, we focused significant attention on her hypoglycemia, which was an immediately life-threatening problem. In this case, any period of fasting for more than four hours was associated with marked hypoglycemia. During the day, our patient prevented severe hypoglycemia via frequent food intake, but prevention of overnight hypoglycemia was more problematic. While under close observation in our hospital, it was clear that continuous administration of either enteral or parental nutrition was effective at preventing overnight hypoglycemia. Possible long-term treatment options are outlined in Table 2. Management options can be significantly different with regard to efficacy, reliability, invasiveness and risk of side effects; with the exception of surgical resection, there are few data to strongly support one modality over another. After thorough discussion of her options, our patient chose frequent overnight food intake in addition to corticosteroid therapy (oral prednisone, 10mg daily). Although this improved our patient’s hypoglycemia, she continued to have periodic hypoglycemia until her death.

## Conclusions

Doege-Potter syndrome is an uncommon paraneoplastic phenomenon associated with SFT characterized by hypoinsulinemic hypoglycemia. In our case, our patient had a malignant ESFT that was not amenable to surgical resection and did not respond to doxorubicin TACE. The diagnosis of Doege-Potter syndrome in our patient was based on the large size of the SFT, low insulin level measured while hypoglycemic, and elevated IGF-II:IGF-I ratio. Her hypoglycemia was managed with daily corticosteroid therapy and frequent nutrient intake, with frequent alarm reminders overnight.

## Consent

Written informed consent was obtained from the patient prior to her death for publication of this case report and accompanying images. A copy of the written consent is available for review by the Editor-in-Chief of this journal.

## Competing interests

The authors declare that they have no competing interests.

## Authors’ contributions

RCS and TAG evaluated previous literature related to solitary fibrous tumors as it related to the case presentation and both were major contributors in writing the manuscript and contributed to the intellectual content of the manuscript. RB and CRM interpreted the hypoglycemia evaluation and both were major contributors in writing the manuscript. GRW analyzed and interpreted the response to chemotherapy and was responsible for critically revising the manuscript. HPC and SLC reviewed the pathology, made critical revisions for content, and provided figures for the manuscript. All authors read and approved the final manuscript.
